# The Optimum Lipid Level for the Juvenile Redclaw Crayfish *Cherax quadricarinatus*: Practical Diets with Soybean Oil as the Lipid Source

**DOI:** 10.1155/2022/2640479

**Published:** 2022-09-20

**Authors:** Chengzhuang Chen, Chang Xu, Xiaolong Yang, Yongyi Jia, Zhimin Gu, Erchao Li

**Affiliations:** ^1^Key Laboratory of Tropical Hydrobiology and Biotechnology of Hainan Province, Hainan Aquaculture Breeding Engineering Research Center, College of Marine Sciences, Hainan University, Haikou, Hainan 570228, China; ^2^Agriculture Ministry Key Laboratory of Healthy Freshwater Aquaculture, Key Laboratory of Fish Health and Nutrition of Zhejiang Province, Key Laboratory of Freshwater Aquaculture Genetic and Breeding of Zhejiang Province, Zhejiang Institute of Freshwater Fisheries, Huzhou 313001, China

## Abstract

As a new species in aquaculture, the lipid nutrition requirement for the juvenile redclaw crayfish *Cherax quadricarinatus* on a dietary basis on a practical formula needs to be evaluated accurately. In this study, the optimum dietary lipid level was explained by analyzing the growth performance, antioxidant state, lipid metabolism, and gut microbiota of *C. quadricarinatus* after an eight-week cultivation trial. Six diets with different soybean oil levels (named L0, L2, L4, L6, L8, and L10) were fed to *C. quadricarinatus* (11.39 ± 0.28 g). The results indicated that the specific growth rate and weight gain of crayfish fed the L4 and L6 diets were significantly higher than those of the other groups (*P* < 0.05). By the analysis of a second-order polynomial regression model according to growth performance (weight gain rate), the optimum lipid level in a practical diet for juvenile *C. quadricarinatus* was 9.67%. The survival, condition factor, and hepatosomatic index of crayfish were not significantly affected by dietary oil levels (*P* > 0.05). As the level of dietary lipids increased, the total antioxidant capacity and glutathione peroxidase activity in serum showed a tendency to rise and then fall and the enzyme activity was highest in crayfish fed the L6 diet. Gut lipase and pepsin activities showed the highest value in crayfish fed the L6 diet. There was no significant difference in acetyl-CoA carboxylase and carnitine palmitoyltransferase-1 contents in crayfish among all the groups (*P* > 0.05). The relative abundance of *Proteobacteria* in the phylum and *Citrobacter* in the genus showed a significant decrease in crayfish of the L10 diet, while the relative abundance of *Firmicutes* in the phylum markedly increased compared to that of the other groups (*P* < 0.05). In summary, the results indicated that the 10.39% (L6 diet) dietary lipid level could induce better growth performance, antioxidant ability, and digestive enzyme activity. Most of the fatty acid composition of muscle is not closely related to the fatty acid composition of the diet. Moreover, the composition and diversity of the gut microbiota of *C. quadricarinatus* were changed by high dietary lipid levels.

## 1. Introduction

Lipids are one of the most important nutrients in the formulation of feed. It is well known that dietary lipids not only are an important source of energy but also provide phospholipids, essential fatty acids, and sterols. These are necessary to maintain the function of physiological processes and to stabilize the biological structure and function of the membrane system of the organism [1, 2]. Lipids contribute to the absorption and transport of vitamins (fat soluble), hormone precursors, vitamin D, and poly-unsaturated fatty acids, such as arachidonic acid [3]. Dietary lipids have also demonstrated a protein sparing effect [4, 5]. In addition, a high-lipid diet in crustaceans may generate peroxidative stress-causing tissue damage and hence increase the risk of excessive release of reactive oxygen species (ROS) [6, 7]. Excessive ROS generation can disrupt the imbalance between oxidative and antioxidant systems, ultimately inducing oxidative stress [7, 8]. Previous studies have indicated that dietary lipid levels could influence growth performance, feed utilization, antioxidant capacity, and immune response in crustaceans such as *Litopenaeus vannamei* [6], *Scylla paramamosain* [3], and *Portunus trituberculatus* [9]. At the same time, optimal dietary lipid levels can reduce the cost of feed production and increase the economic efficiency of agricultural production [10].

As an important physical and biological barrier for aquatic animals, the gut is an essential organ that aids in nutrition absorption, digestion, immunity, and disease prevention [11]. The gut includes billions of bacteria that perform a variety of important activities for the host, including nutrition absorption, energy balance maintenance, and immune response promotion [12, 13]. The gut bacterial community participates in a variety of physiological metabolic activities, such as lipid storage, energy balancing, and bile acid synthesis [14]. In aquatic animal studies, diets with different lipid sources can remodel the composition pattern of the gut microbiota of *L. vannamei* [15, 16]. Dietary lipid levels could influence the composition of the gut microbiota in *P. trituberculatus* [17]. However, studies on the effect of dietary lipid levels on the gut microbiota of redclaw crayfish *Cherax quadricarinatus* are still lacking. Therefore, the present research used gut microbiota, an evaluation system, to explain the positive response of the gut microbiota to nutrient intake.

*C. quadricarinatus* is native to the northern tropics of Australia and southeastern Papua New Guinea and is widely farmed in many tropical and subtropical countries [18–20]. To facilitate the development of intensive farming systems, it is necessary to develop high-quality practical feed formulations and obtain information about the nutritional requirements of crayfish. Although several previous studies have determined the optimal lipid requirements of *C. quadricarinatus*, there are relatively few studies on the impact of dietary lipid levels on biochemical parameters and gut microbiota [21–23]. In our previous studies, it was demonstrated that soybean oil can be the optimum lipid source choice for juvenile *C. quadricarinatus* [24]. On this basis, soybean oil was a supplement in the practical diet as the main oil source for juvenile *C. quadricarinatus*. Therefore, this study is aimed at assessing the effect of dietary lipid levels on the growth performance, antioxidant capacity, digestive capacity, lipid metabolism, and gut microbiota of juvenile red crayfish to assess the lipid requirements of red crayfish more accurately.

## 2. Materials and Methods

### 2.1. Experimental Diets

Six isonitrogenous (37% crude protein) practical diets were formulated containing 4.32%, 6.30%, 8.33%, 10.39%, 12.23%, and 13.97% crude lipids (named L0, L2, L4, L6, L8, and L10, respectively). The formulation and proximate composition of the experimental diets are presented in Table 1. The main dietary protein sources included fish meal, soybean meal, and cottonseed meal. Soybean oil was used as the main oil source. For the diet production procedure, refer to our previous study [24]. The composition of fatty acids in the experimental diets is presented in Table 2.

### 2.2. Crayfish Rearing and Feeding Trial

Redclaw crayfish were obtained from a local breeding factory (Chengmai, Hainan, China) and acclimatized to concrete pond conditions with commercial feed for a week. Healthy crayfish (initial weight 11.39 ± 0.28 g) were selected and randomly assigned to 24 net cages (1.0 mL × 0.5 mW × 1 mH). In the experiment, crayfish were set up in six groups with four replicates. Each net cage contained 15 crayfish. These nets were neatly placed in the concrete pond, and polyethylene pipes were placed at the bottom of the nets as shelters for the crayfish. For experimental farming conditions and management during farming, please refer to our previous study [24]. Briefly, water temperature, pH, and dissolved oxygen were monitored daily and recorded as 24–27°C, 7.8–8.2, and >4 mg/L, respectively. Regularly remove the feces and uneaten feed from the net cage, and update about 60% of the water every three days to maintain a normal water quality environment. The rearing trial was conducted for eight weeks with daily quantitative feeding (4% weight of crayfish) at 10 : 00 and 18 : 00.

### 2.3. Sampling Collection

After the eight-week feeding trial, all crayfish were deprived of feed for 24 h before sampling. The total numbers and final weights of crayfish were measured to calculate the survival, specific growth rate (SGR), weight gain rate (WGR), condition factor (CF), and hepatosomatic index (HSI). In addition, the sampling strategies for the hemolymph, gut, and hepatopancreas were described in detail in our previous study [24]. All experimental procedures, animals, and designs were performed and approved following the guidelines of the Hainan University Institutional Animal Use and Care Committee (HNUAUCC-2020-00004).

### 2.4. Whole-Body Composition Analysis

Before tissue sampling, six crayfish in each experimental group were sampled for whole-body composition analysis. The proximate composition of diets and the whole-body composition of crayfish were analyzed using standard procedures. Briefly, the parameters mainly included crude protein, moisture, crude lipid, and ash. The details of the experimental procedure were as described in a previous study [24].

### 2.5. Fatty Acid Analysis in Diets and Muscle

The fatty acid profile in the experimental diet and muscle of juvenile crayfish was determined according to the method in a previous study [24]. Briefly, approximately 0.1 g of the lyophilized sample was esterified in the same manner and the fatty acid methyl esters were separated into microbottles and stored. Fatty acid contents were quantified and analyzed by gas chromatography-mass spectrometry (GC-MS). The relative composition of fatty acids was calculated through the analysis of the ratio of the considered subpeak area to the total peak area.

### 2.6. Biochemical Analysis

Hepatopancreases and gut samples were homogenized with precooled sterile 0.9% saline solution at a ratio of 10% as *m* (tissue, g) : *v* (saline solution, mL). The homogenates were centrifuged at 3500 rpm for 10 min at 4°C. The supernatants were collected and analyzed by commercial kits for biochemical parameters in a microplate reader (BioTek Epoch, USA). Serum antioxidant capacity was assessed by the following indicators: superoxide dismutase (SOD) (A001-3-2) activity, glutathione peroxidase (GSH-PX) (A005-1-2) activity, malondialdehyde (MDA) (A003-1-2) content, and total antioxidant capacity (T-AOC) (A015-2-1). All these values were obtained by using commercial kits (Jiancheng, Nanjing, China). Similarly, the contents of carnitine palmitoyltransferase-1 (CPT-1), acetyl-CoA carboxylase (ACC), total protein (TP) (A045-2-2), total cholesterol (TC) (A111-1-1), and triacylglycerol (TG) (A110-1-1) in hepatopancreases were tested by corresponding commercial kits (Jiancheng, Nanjing, China). The activities of gut pepsin, lipase, and amylase were tested by commercial kits with the types A080-1-1, A054-2-1, and C016-1-1, respectively (Jiancheng, Nanjing, China).

### 2.7. Gut Microbiota Analysis

Gut samples from the L0, L6, and L10 groups were selected for gut microbiota analysis based on growth performance and physiological status after eight weeks of cultivation. Total bacterial DNA from the gut samples was extracted using a DNA extraction kit (Omega Bio-Tek, Norcross, GA, USA). The DNA integrity was confirmed using 1.0% agarose gel electrophoresis, and the DNA concentration and quality were quantified using a NanoDrop 2000 spectrophotometer (Thermo Fisher Scientific, Waltham, MA, USA). The V3 and V4 hypervariable region products of the 16S rRNA gene were amplified by the barcoded fusion primers 338 F (5′-ACTCCTACGGGAGGCAGCA-3′) and 806 R (5′-GGACTACHVGGGTWTCTAAT-3′). The volume and PCR program were carried out using a published procedure [25]. The Illumina MiSeq PE300 platform was used to generate paired-end reads from purified PCR products. The raw sequences have been submitted to the National Center for Biotechnology Information (NCBI) (https://submit.ncbi.nlm.nih.gov) database (BioProject ID: PRJNA699999).

Raw sequence data were filtered using QIIME (version v1.9.1), and the effective tags were clustered into operational taxonomic units (OTUs) from high-quality sequences with 97% similarity by using UPARSE (version 7.0.1090). Each representative sequence selected from each OTU can be annotated by taxonomic information according to the mothur and SILVA SSUrRNA database [26]. The alpha diversity indices, including the ACE, Chao 1, Simpson, and Shannon indices, were calculated by using QIIME. A Venn diagram was constructed to identify the shared and unique OTUs. Beta diversity analysis using QIIME assessed the bacterial community structure based on the unweighted UniFrac distance metric, mainly including analysis of similarities (ANOSIM), nonmetric multidimensional scaling analysis (NMDS), and Adonis. Linear discriminant analysis (LDA) effect size (LEfSe) analysis was used to identify bacterial taxa that differed between the two experimental groups. The function of the gut microbiota was predicted using the PICRUSt bioinformatics program, with the Kyoto Encyclopedia of Genes and Genomes (KEGG) indicating the forecasted functional pathologies at different KEGG levels [27].

### 2.8. Statistical Analysis

Statistical analysis by one-way analysis of variance (ANOVA) and Duncan's multiple range tests was carried out to analyze the significant differences among all the groups. For the KEGG pathways, an independent samples *t*-test was used to compare significant differences between the two experimental groups. *P* < 0.05 was regarded as a significant difference. All the data are described as the means ± SE (standard error). The Pearson correlation coefficients between genera with abundance in the top 50 were determined. A correlation coefficient matrix diagram was drawn in the bioinformatics platform (https://www.bioinformatics.com.cn). In addition, the optimal lipid supplementation level in the diet for juvenile redclaw crayfish was determined using a second-order polynomial regression model based on WG. All statistical analyses were performed with SPSS 25.0 software (IBM, version 25.0, Armonk, USA).

## 3. Results

### 3.1. Growth Performance

**Figure 1:**
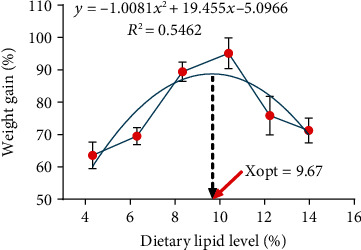
The relationship between weight gain (WG) (%) and the different dietary lipid levels. Xopt represents the optimal dietary lipid level for the maximum WG of juvenile *C. quadricarinatus*. Each point in the picture represents the mean value of four replicates.

Survival ranged from 73.3% to 75.0% and showed no significant difference among all diets (*P* > 0.05). The WG and SGR of crayfish fed the L4 and L6 diets were significantly higher than those fed the other diets (*P* < 0.05). Crayfish fed the L6 diet showed the best growth performance, although there was no significant difference from the L4 diet (*P* > 0.05). Different dietary lipid levels had no significant influence on crayfish survival, CF, or HSI (*P* > 0.05) (Table 3). Through the second-order polynomial regression model analysis based on WG, 9.67% was the optimal dietary lipid content to obtain maximum growth performance for the juvenile *C. quadricarinatus* (Figure 1).

### 3.2. Whole-Body Proximate Composition

The whole-body proximate compositions of crayfish fed diets with different lipid levels are presented in Table 4. There was no significant difference in the contents of crude protein, crude lipid, moisture, and ash of crayfish among all the groups (*P* > 0.05).

### 3.3. Composition of Fatty Acids in Diets and Muscle

**Figure 2:**
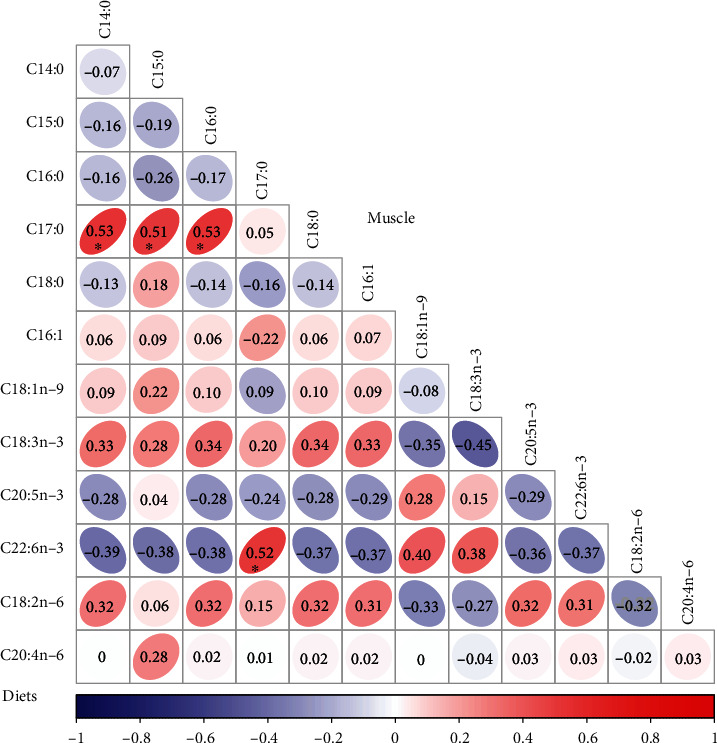
The correlation between the fatty acid compositions in tail muscle and diets by Pearson correlation matrix analysis. The color scale indicates the correlation value from −1 to 1. The red color indicates a positive correlation. The blue color indicates a negative correlation. ^∗^Significant difference (*P* < 0.05).

The dietary composition of fatty acids is presented in Table 2. The percentages of total monounsaturated fatty acids (∑MUFA) and total polyunsaturated fatty acids (∑PUFA) in the L0 diet were significantly higher than those in crayfish fed the other diets (*P* < 0.05). The ratio of n-3/n-6 in the L0 diet was also higher than those in other diets (*P* < 0.05). The muscle fatty acid composition in juvenile crayfish fed diets with different lipid levels is shown in Table 5. These results indicated that no significant difference was found in fatty acid composition in the muscle of crayfish fed diets with different lipid levels (*P* > 0.05). The matrix analysis of the Pearson coefficient between the fatty acid composition of the diets and the tail muscle is shown in Figure 2. The results indicated that C14:0, C15:0, C16:0, and C22:6n-3 in muscle were significantly positively correlated with C17:0 in diets (*P* < 0.05). Furthermore, most of the fatty acids in muscle were not closely related to the dietary fatty acid composition.

### 3.4. Antioxidant Capacity

**Figure 3:**
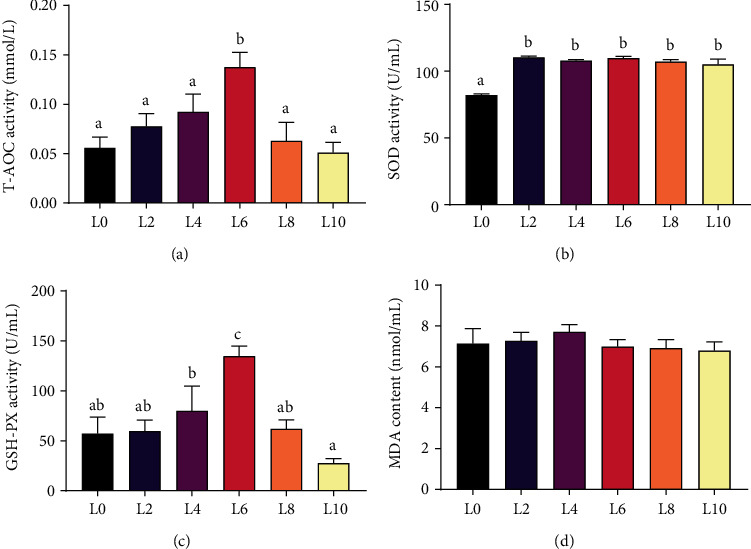
The activities of T-AOC (a), SOD (b), GSH-PX (c), and MDA (d) in the serum of *C. quadricarinatus* fed diets with different lipid levels. All data are expressed as the mean ± SE (*n* = 6). Different letters indicate significant differences among experimental groups (*P* < 0.05).

With the increase in dietary lipid level, the activities of T-AOC and GSH-PX in serum showed an increasing change and the highest activities were found in the L6 diet (Figures 3(a) and 3(c)) (*P* < 0.05). The serum activity of SOD in crayfish in the L0 diet was significantly lower than that in other groups (Figure 3(b)) (*P* < 0.05). There was no significant difference in the serum MDA concentration of crayfish among all the groups (Figure 3(d)) (*P* > 0.05).

### 3.5. Digestive Enzyme Activity

**Figure 4:**
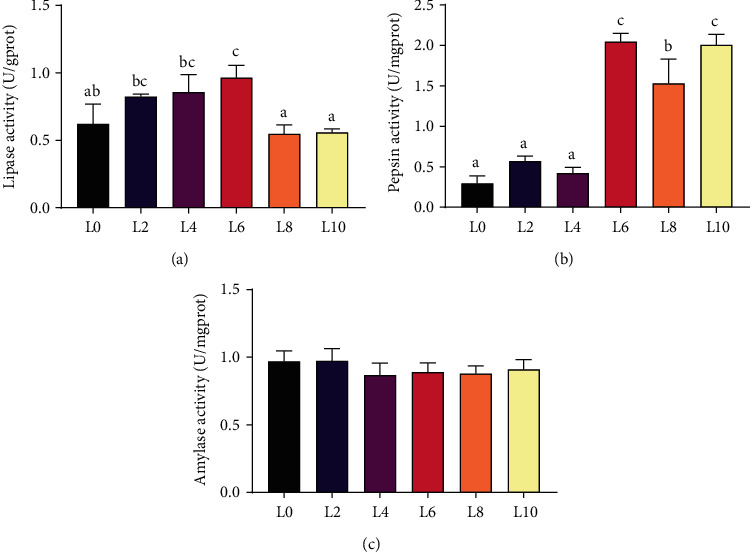
The activities of lipase (a), pepsin (b), and amylase (c) in the gut of *C. quadricarinatus* fed diets with different lipid levels. All data are expressed as the mean ± SE (*n* = 6). Different letters indicate significant differences among experimental groups (*P* < 0.05).

The gut lipase of crayfish showed the highest activity in the L6 diet and was significantly higher than the activities in the L0, L8, and L10 diets (Figure 4(a)) (*P* < 0.05). The activities of pepsin in the gut of crayfish in the L0, L2, and L4 diets were significantly lower than those in other groups, with the highest enzyme activity in the L6 diet (Figure 4(b)) (*P* < 0.05). No significant difference was found in the amylase activity of crayfish fed diets with different lipid levels (Figure 4(c)) (*P* > 0.05).

### 3.6. Lipid Metabolism

**Figure 5:**
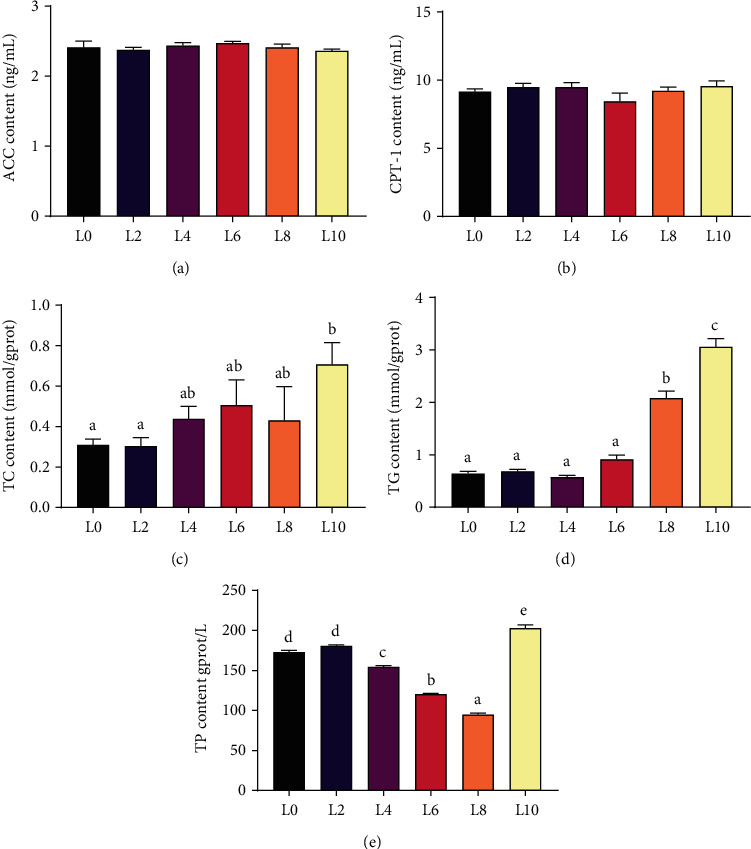
The contents of ACC (a), CPT-1 (b), TG (c), TC (d), and TP (e) in the hepatopancreas of *C. quadricarinatus* fed diets with different lipid levels. All data are expressed as the mean ± SE (*n* = 6). Different letters indicate significant differences among experimental groups (*P* < 0.05).

Dietary lipid levels had no significant influence on the contents of ACC and CPT-1 in the hepatopancreas of crayfish (Figures 5(a) and 5(b)) (*P* > 0.05). With the increase in dietary lipid levels, TC and TG contents showed an overall increasing trend and TG content in the L10 diet was significantly higher than in other groups (Figures 5(c) and 5(d)) (*P* < 0.05). The lowest TP content was observed in crayfish fed the L8 diet, and the highest value was observed in crayfish fed the L10 diet (Figure 5(e)) (*P* < 0.05).

### 3.7. Gut Microbiota Composition and Diversity

**Figure 6:**
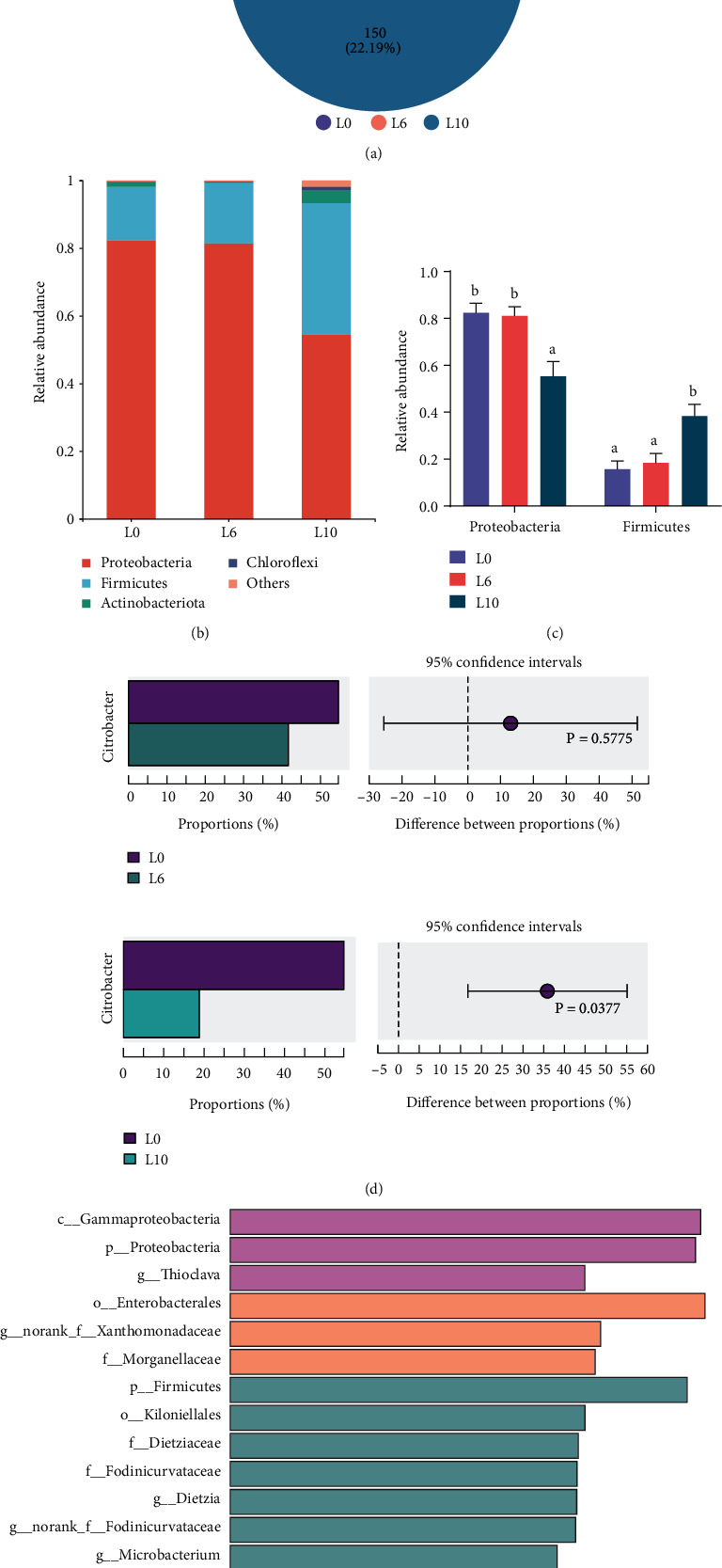
Differences in bacterial community composition in *C. quadricarinatus* fed diets with different lipid levels. (a) The numbers of shared and unique OTUs. (b) The relative abundance of gut microbiota in *C. quadricarinatus* by phylum. (c) Comparisons of the relative abundance of the major bacteria in *C. quadricarinatus* at the phylum level. (d) The relative abundance ratio at the genus level. The middle shows the difference between proportions of relative abundance in the 95% confidence interval, and *P* < 0.05 represents a significant difference. (e) Bacterial taxa differentially displayed in the gut of *C. quadricarinatus* raised were identified by LEfSe using an LDA score threshold of >3.5. Data are expressed as the mean ± SE (*n* = 4). Different letters indicate significant differences among experimental groups (*P* < 0.05).

The gut microbiota of crayfish in the L0, L6, and L10 diets was analyzed. The total number of sequences was 667,942 from 12 samples in the three groups. The 251 OTUs were shared by the three groups. The numbers of unique OTUs in the L0, L6, and L10 diets were 36, 27, and 150, respectively (Figure 6(a)). The most dominant phyla were *Proteobacteria*, *Firmicutes*, and *Actinobacteria* in all samples (Figure 6(b)). At the phylum level. The relative abundance of *Proteobacteria* was significantly lower in crayfish fed the L10 diet than in those fed the L0 and L6 diets. The relative abundance of *Firmicutes* was higher than that in the L0 and L6 diets (Figure 6(c)). At the genus level, the abundance of *Citrobacter* in the L10 diet was significantly decreased compared with that in the L0 diet (Figure 6(d)) (*P* < 0.05). Some bacterial taxa at different taxonomic levels were highly enriched in the three groups, according to LEfSe analyses (Figure 6(e)).

**Figure 7:**
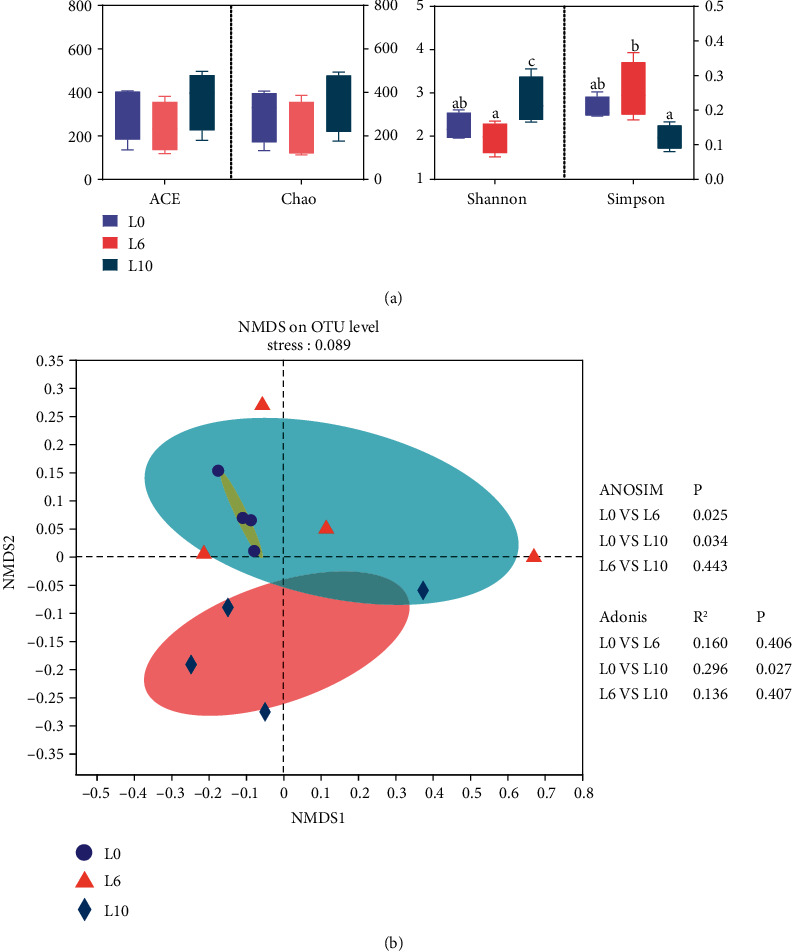
Diversity of the bacterial community in *C. quadricarinatus* fed diets with different lipid levels. (a) Alpha diversity. (b) Beta diversity. All data are expressed as the mean ± SE (*n* = 4), and *P* < 0.05 was considered significantly different.

No significant difference in the Chao1 and ACE estimators (Richness estimate) was observed in crayfish among all three groups, while the Simpson index was significantly decreased and the Shannon index was significantly increased in the L10 diet (Figure 7(a)). NMDS analysis showed that the gut microbiota was significantly clustered in each group, with the L10 diet showing a more significant separation than the other two groups (Figure 7(b)). ANOSIM and Adonis analysis further confirmed the results of NMDS analysis (*P* = 0.034; *P* = 0.027).

### 3.8. Functional Prediction and Co-Occurrence Patterns of the Gut Microbiota

**Figure 8:**
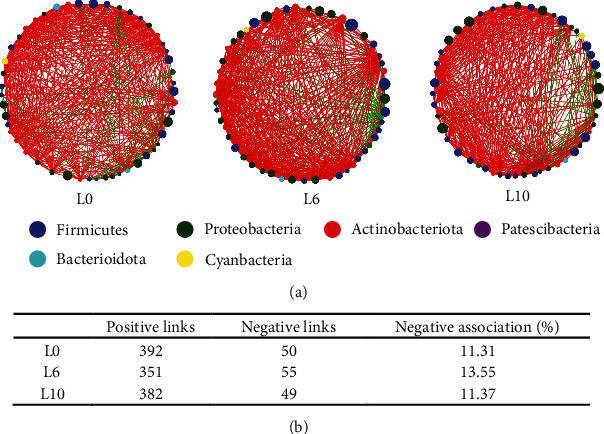
Correlation-based network analysis of the bacterial community in *C. quadricarinatus* fed diets with different lipid levels. (a) Each node represents one genus, its colors represent the different phylum levels of bacteria, and its size was proportional to the number of connections (degree). The green edge indicates a negative interaction between two individual nodes. The red edge indicates a positive interaction between two individual nodes. (b) Interspecies interaction types and the ratio of negative interactions in the ecological network.

PICRUSt was used to predict the bacterial gene function from 16S rRNA sequencing data. The KEGG level 3 pathways with significant differences are listed in Table 6. KEGG pathways related to metabolism, cellular transformation, organic systems, and environmental information processing showed significant changes with the increase in the dietary lipid level. At KEGG pathway level 3, retinol metabolism, cytoskeleton proteins, antigen processing and presentation, bacterial toxins, and transporters were significantly increased in the L10 diet. An interspecies interaction network was established to assess the influence of dietary lipid levels on interspecies interactions of the gut microbiota in crayfish. The correlation network graph revealed that crayfish fed the L6 diet had closer and more complex interspecies interactions than crayfish in the other groups (Figure 8(a)). In the L6 diet, the gut microbiota of crayfish presented a higher ratio of negative association than in the other groups (Figure 8(b)).

## 4. Discussion

In general, the true lipid requirement of crustaceans cannot be clearly defined because it is influenced by a variety of factors, including species differences, growth stage, and cultural environment. As a macronutrient, the highest weight gain is achieved by adding 5–6% levels to the diet of crustaceans and the optimal level of dietary lipid addition is usually 5% to 8% [28]. In the present study, the growth performance of *C. quadricarinatus* was significantly influenced by different dietary lipid levels, which is similar to what has been observed in other typical aquatic animals [29, 30]. In our study, crayfish showed the best growth performance in the L6 diet. In crustaceans, diets with low lipid levels cannot provide enough essential fatty acids to meet the physiological functional requirements for growth and development [7]. In contrast, diets with high lipid levels result in reduced dietary intake and digestive enzyme activity in crustaceans and affect the utilization of lipids and other essential nutrients [31]. In this study, the growth performance of crayfish improved and then decreased with increasing dietary lipid levels. From the perspective of energy utilization, the possible reason is that the energy supplied by the high-lipid diet has exceeded the demand, which results in metabolic imbalance and reduces feed consumption and the use of other nutrients [17, 32]. Changes in growth performance are closely related to organism digestive enzymes. In this study, L6 diet intake significantly increased the lipase activity of *C. quadricarinatus* and the results corroborated the improvement in growth performance. Therefore, the appropriate dietary lipid level contributes to the growth performance and digestibility of *C. quadricarinatus*.

Many studies have shown that the whole-body lipid content increases with increasing dietary lipid levels and excess lipids in the diet may lead to excess fat deposits in the tissues [3, 22, 33]. In this study, the crude lipid content of crayfish did not show significant differences among all the groups. Different dietary lipid levels did not significantly affect the contents of moisture, crude protein, and ash. These results were similar to the findings of many previous studies [34, 35]. It is widely acknowledged that the fatty acid composition in the tissues of crustaceans corresponds to the fatty acid pattern in the corresponding diet [3, 5, 36, 37]. In this study, with the increase in dietary lipids, there was no significant difference in the fatty acid composition in the tail muscle of crayfish among all the groups. Fatty acids in muscle tissue were not positively correlated with dietary fatty acid composition except for C14:0, C15:0, C16:0, and C22:6n-3 in muscle and C17:0 in the diet. These results differ from a previous report, probably because the fatty acid composition in tissues is the result of the complex interrelationships among various physiological metabolic processes (e.g., dietary fatty acid intake, transfer, and oxidative breakdown) [24].

There is a balance between ROS production and removal in organisms under normal physiological conditions. Excessive ROS can cause damage to the organism, while the increased activity of antioxidant enzymes (such as GSH-PX and SOD) is the main mechanism to eliminate ROS and protect cells from damage caused by lipid peroxidation [38–40]. The activity of T-AOC can reflect the status of antioxidant defense in cells, and the MDA content can reflect the level of lipid peroxidation in organisms [39, 40]. In this study, the highest GSH-PX activity and T-AOC were both observed in the serum of crayfish in the L6 diet treatment and the lowest SOD activity was observed in the L0 diet, while the MDA content showed no significant difference among the different dietary groups. Previous research has demonstrated that an oxidative and antioxidant system could interfere with the balance of overproduction, ultimately leading to oxidative stress [8, 41]. Therefore, appropriate dietary lipid levels contribute to the body's capacity to endure oxidative stress.

Diets with different lipid levels can affect lipid metabolism in aquatic animals [30, 42]. The hepatopancreas of crustaceans has multiple functions and is the main site of lipid metabolism. ACC is the rate-limiting enzyme in fatty acid synthesis [43, 44]. CPT-1 catalyzes the conversion of the fatty acid-coenzyme A complex into the fatty acid-carnitine complex for entry into the mitochondrial matrix and is considered to be a major regulator of the oxidation of long-chain fatty acids [45]. The present results showed that diets with different lipid levels did not affect the contents of ACC and CPT-1 in the hepatopancreas of crayfish. It is important to note that lipid metabolism involves many important enzymes and transcription factors and is a complex process [46]. Several studies have suggested that the activity of ACC and CPT-1 in the hepatopancreas increases with increasing dietary lipid levels in largemouth bass (*Micropterus salmoides*) [30]. Overall, our study showed that diets with different lipid levels had no effects on the lipid metabolic activity of crayfish but the molecular mechanisms underlying the effects of diets with different lipid levels on crayfish lipid metabolism need further study.

For crustaceans, the stability of the gut microbiota is one of the most important health parameters and the composition of the gut microbiota changes according to metabolic and immune conditions [47]. Shannon and Simpson are used to defining species richness and evenness, whereas ACE and Chao 1 are abundance-based estimators of species richness [48, 49]. No significant difference was found in the Chao 1 and Ace indices in these three groups. L10 diet ingestion can significantly improve the Shannon index and reduce the Simpson index in the gut of crayfish. These results indicated that diets with different lipid levels may have more significant effects on the species evenness of gut microbiota than on the species richness. According to NMDS, ANOSIM, and Adonis analysis, the microbial community structure in crayfish fed the L10 diet was significantly changed compared with that in crayfish fed the L0 diet, which indicated that the high-lipid diet had a strong impact on the overall pattern and structure of the gut microbiota of crayfish.

The dominant phyla in the gut of crayfish were *Proteobacteria*, *Firmicutes*, and *Actinobacteria*, which was consistent with previous studies [50, 51]. In the high-lipid dietary treatment, the relative abundance of *Proteobacteria* was significantly lower than that in the other groups, while that of *Firmicutes* was significantly higher than that in the other groups. *Proteobacteria* is a common phylum of bacteria in the gut of aquatic animals. Some species of *Firmicutes* are members of lactic acid bacteria and are involved in the degradation of polysaccharides in the body, which is a major factor in obesity [52]. Some studies have found that changes in *Proteobacteria* may be a sign of an imbalance in the gut microbiota [53]. Therefore, the relative abundance of some potentially diseasing in the host may alter different levels of dietary lipids and indirectly affect aquatic animal growth and health. However, the mechanism of dietary lipid levels on the changes in crayfish gut microbiota still needs to be investigated deeply.

The PICRUSt results revealed that compared to the L6 diet, the L10 diet significantly increased retinol metabolism, cytoskeleton proteins, antigen processing and presentation, bacterial toxins, and transporters. This result indicated that high-lipid diets can lead to changes in various functional pathways due to fluctuations in host-microbial diversity and major bacteria at the phylum level. PICRUSt can only be used to speculate gut bacterial functions. More study is needed to validate the accuracy of gut bacterial function prediction using meta-genomic analysis [11]. The complex microbiota does not exist in isolation but creates a complex ecological network of interactions, such as predation, competition, and mutualism, through various kinds of interactions [54]. Previous research revealed that a high proportion of negative links indicates a more complex interaction between species and a more stable microbiota community [55–57]. In this study, the ratio of negative connections in the bacterial network of crayfish in the L6 diet was higher than those in other experimental diets, indicating that the L6 diet had a more stable microbiota community. Therefore, appropriate levels of dietary lipids could increase bacterial network complexity.

In conclusion, an appropriate dietary lipid could improve growth performance, antioxidant ability, digestive enzyme activity, and the stability of gut microbiota in juvenile *C. quadricarinatus*. The deposition effect of dietary fatty acids on the muscle of crayfish is not ideal. According to second-order polynomial regression model analysis based on growth performance (weight gain rate), the optimum lipid levels in a practical diet for juvenile *C. quadricarinatus* is 9.67% based on the lipid source of soybean oil.

## Figures and Tables

**Table 1:** Ingredient formulation (g/kg dry matter) and proximate composition (%) of the six experimental diets for juvenile *C. quadricarinatus*.

Ingredients	Experimental diets					
	L0	L2	L4	L6	L8	L10
Fish meal	300	300	300	300	300	300
Soybean meal	150	150	150	150	150	150
Cottonseed meal	150	150	150	150	150	150
Wheat starch	200	200	200	200	200	200
Soybean oil	0	20	40	60	80	100
Choline chloride^a^	10	10	10	10	10	10
Sodium carboxymethylcellulose	25	25	25	25	25	25
Cholesterol^a^	5	5	5	5	5	5
Soybean lecithin^b^	10	10	10	10	10	10
*α*-Cellulose	110	90	70	50	30	10
Butylated hydroxytoluene	0.05	0.05	0.05	0.05	0.05	0.05
Vitamin premix ^c^	20	20	20	20	20	20
Mineral premix ^d^	20	20	20	20	20	20
Total	1000	1000	1000	1000	1000	1000
Analyzed proximate composition (%)						
Crude protein	36.60	36.92	37.20	37.23	37.06	37.10
Crude lipid	4.32	6.30	8.33	10.39	12.23	13.97
Moisture	9.17	10.00	10.22	9.67	9.83	10.17
Ash	7.66	7.60	7.47	7.68	7.29	7.69

^a^Sangon Biotech Ltd., Shanghai, China. ^b^Shanghai Taiwei Ltd., Shanghai, China. ^c^Vitamin premix (per 100 g premix): thiamin, 0.05 g; riboflavin, 0.3 g; pyridoxine, 0.1 g; cyanocobalamin, 0.03 g; ascorbic acid (35%), 10 g; alpha tocopherol (50%), 5 g; menadione, 0.2 g; inositol, 0.5 g; nicotinamide, 0.5 g; cholecalciferol (500000 IU/g), 0.08 g; retinol palmitate (500000 IU/g), 0.05 g; folic acid, 0.02 g; biotin, 0.005 g; choline chloride, 10 g; pantothenic acid, 0.5 g. All ingredients were filled with *α*-cellulose to 100 g. ^d^Mineral premix (per 100 g premix): KCl, 2.8 g; MgSO4, 7.1 g; ZnSO_4_, 2 g; MnSO_4_, 0.162 g; CuSO_4_.5H_2_O, 0.06 g; Ca(IO_3_)_2_, 0.02 g; CoCl_2_.6H_2_O 0.01 g, NaH_2_PO_4_, 21.5 g; CaHPO_4_.2H_2_O, 25 g; CaCO_3_, 18 g; Na_2_SeO_3_, 0.025 g; FeSO_4_.H_2_O, 1.0 g; KH_2_PO_4_, 21.6 g. All ingredients were diluted with *α*-cellulose to 100 g.

**Table 2:** Fatty acid composition in the six experimental diets (% of total fatty acids).

Parameters	Experimental diets					
	L0	L2	L4	L6	L8	L10
C14:0	5.59 ± 0.17^d^	2.56 ± 0.10^c^	1.24 ± 0.06^b^	1.21 ± 0.06^b^	1.07 ± 0.01^ab^	0.89 ± 0.01^a^
C15:0	0.45 ± 0.004^b^	0.22 ± 0.002^ab^	0.12 ± 0.001^a^	0.11 ± 0.001^a^	0.09 ± 0.002^a^	0.31 ± 0.002^ab^
C16:0	24.21 ± 0.06^a^	17.13 ± 0.09^b^	14.14 ± 0.03^c^	14.12 ± 0.23^c^	13.30 ± 0.19^d^	13.06 ± 0.04^d^
C17:0	0.21 ± 0.01^ab^	0.09 ± 0.01^a^	0.29 ± 0.002^b^	0.13 ± 0.08^ab^	0.11 ± 0.07^a^	0.16 ± 0.06^ab^
C18:0	7.72 ± 0.03^d^	5.62 ± 0.03^c^	4.77 ± 0.003^b^	4.70 ± 0.08^b^	4.49 ± 0.06^a^	4.45 ± 0.01^a^
∑SFA^a^	38.18 ± 0.26^d^	25.63 ± 0.21^c^	20.56 ± 0.09^b^	20.28 ± 0.37^b^	19.06 ± 0.29^a^	18.88 ± 0.12^a^
C16:1	4.17 ± 0.02^e^	1.94 ± 0.01^d^	1.07 ± 0.01^c^	1.01 ± 0.05^c^	0.79 ± 0.04^b^	0.69 ± 0.01^a^
C18:1n-9	20.87 ± 0.08^a^	24.61 ± 0.05^b^	26.71 ± 0.07^c^	26.20 ± 0.15^c^	26.83 ± 0.13^c^	27.02 ± 0.04^c^
∑MUFA^b^	25.04 ± 0.08^a^	26.55 ± 0.04^b^	27.78 ± 0.10^c^	27.21 ± 0.10^c^	27.62 ± 0.09^c^	27.71 ± 0.03^c^
C18:3n-3	0.25 ± 0.003^a^	0.21 ± 0.03^a^	4.46 ± 0.002^b^	4.56 ± 0.04^c^	4.67 ± 0.10^d^	4.74 ± 0.01^d^
C20:5n-3	1.45 ± 0.02^d^	0.70 ± 0.01^c^	0.39 ± 0.003^b^	0.38 ± 0.04^b^	0.28 ± 0.025^a^	0.26 ± 0.003^a^
C22:6n-3	1.14 ± 0.01^d^	0.54 ± 0.01^c^	0.28 ± 0.004^b^	0.26 ± 0.02^b^	0.20 ± 0.02^a^	0.18 ± 0.01^a^
∑n-3PUFA^c^	2.84 ± 0.02^b^	1.45 ± 0.04^a^	5.13 ± 0.01^c^	5.20 ± 0.03^c^	5.17 ± 0.05^c^	5.18 ± 0.01^c^
C18:2n-6	21.81 ± 0.11^a^	37.08 ± 0.18^b^	42.82 ± 0.08^c^	43.61 ± 0.47^c^	44.86 ± 0.32^d^	45.31 ± 0.09^d^
C20:4n-6	0.25 ± 0.002^d^	0.13 ± 0.02^c^	0.07 ± 0.001^b^	0.06 ± 0.006^b^	0.05 ± 0.01^a^	0.05 ± 0.002^a^
∑n-6PUFA^d^	22.06 ± 0.11^a^	37.21 ± 0.18^b^	42.88 ± 0.08^c^	43.67 ± 0.47^c^	44.91 ± 0.31^d^	45.35 ± 0.09^d^
∑PUFA^e^	24.90 ± 0.09^a^	38.66 ± 0.18^b^	48.02 ± 0.08^c^	48.88 ± 0.45^c^	50.08 ± 0.26^d^	50.53 ± 0.09^d^
n-3/n-6PUFA^f^	0.13 ± 0.001^d^	0.04 ± 0.001^a^	0.12 ± 0.001^c^	0.12 ± 0.001^c^	0.12 ± 0.002^c^	0.11 ± 0.001^b^

^a^∑SFAs: saturated fatty acids: C14:0, C15:0, C16:0, C17:0, and C18:0. ^b^∑MUFA: monounsaturated fatty acids: C16:1 and C18:1n-9. ^c^∑n-3 PUFA: omega 3 polyunsaturated fatty acids: C18:3n-3, C20:5n-3, and C22:6n-3. ^d^∑n-6 PUFA: omega 6 polyunsaturated fatty acids: C18:2n-6 and C20:4n-6. ^e^∑PUFA: polyunsaturated fatty acids: C18:3n-3, C20:5n-3, C22:6n-3, C18:2n-6, and C20:4n-6. ^f^n-3/n-6 PUFA: omega 3 polyunsaturated fatty acids: omega 6 polyunsaturated fatty acids. Data are reported as the mean ± SE of three replicates (*n* = 3). Data with different letters were significantly different (*P* < 0.05) among all the groups.

**Table 3:** Growth performance of juvenile *C. quadricarinatus* fed diets with different lipid levels.

Parameters	Experimental diets					
	L0	L2	L4	L6	L8	L10
Survival (%)	73.33 ± 2.72	75.00 ± 3.19	75.00 ± 3.19	73.33 ± 2.72	75.00 ± 3.19	75.00 ± 3.19
IBW	10.19 ± 0.15	11.46 ± 0.21	12.17 ± 0.20	9.34 ± 0.94	11.90 ± 0.16	13.28 ± 0.89
FBW	16.66 ± 0.59^a^	19.43 ± 0.55^bc^	23.06 ± 0.59^e^	18.22 ± 0.38^ab^	20.94 ± 0.84^cd^	22.73 ± 0.58^de^
WGR (%)	63.57 ± 4.10^a^	69.51 ± 2.60^a^	89.44 ± 2.97^b^	95.14 ± 4.76^b^	75.87 ± 5.97^a^	71.26 ± 3.86^a^
SGR (% day−^1^)	0.86 ± 0.44^a^	0.94 ± 0.03^a^	1.14 ± 0.03^b^	1.19 ± 0.04^b^	1.01 ± 0.06^a^	0.96 ± 0.04^a^
CF (%)	2.40 ± 0.11	2.33 ± 0.10	2.29 ± 0.04	2.43 ± 0.10	2.43 ± 0.23	2.50 ± 0.06
HSI (%)	5.15 ± 0.25	5.22 ± 0.45	5.58 ± 0.39	6.11 ± 0.48	5.55 ± 0.40	5.91 ± 0.52

IBW: initial body weight; FBW: final body weight; WGR: weight gain rate; SGR: specific growth rate; CF: condition factor; HSI: hepatosomatic index. All data are expressed as the mean ± SE (*n* = 4). Different letters indicate significant differences between groups (*P* < 0.05). Survival rate (%) = *S*_0_/*S*i × 100. WGR (%) = (*W*_t_ − *W*_0_)/*W*_0_ × 100. SGR (% · day^−1^) = [ln*W*_t_–ln*W*_0_]/*T* × 100. CF (%) = *W*_t_/*L*^3^ × 100. HSI (%) = (*W*_h_/*W*_t_) × 100, where *S*_0_ and *S*i are the initial crayfish numbers and final crayfish numbers, respectively. *W*_t_, *W*_0_, and *W*_h_ (g) are the means of the final wet body weight, initial wet body weight, and wet hepatopancreatic weight, respectively. *T* and *L* are the duration (days) of the experiment and body length (cm), respectively.

**Table 4:** Proximate composition (%, wet weight) of juvenile *C. quadricarinatus* fed diets with different lipid levels.

Parameters	Experimental diets					
	L0	L2	L4	L6	L8	L10
Crude protein	13.85 ± 0.24	14.26 ± 0.22	13.70 ± 0.31	13.93 ± 0.58	14.36 ± 0.25	14.05 ± 0.24
Crude lipid	2.15 ± 0.42	2.46 ± 0.30	2.12 ± 0.50	2.01 ± 0.69	2.16 ± 0.68	2.14 ± 0.55
Ash	9.08 ± 2.31	8.94 ± 1.56	8.9 7 ± 2.22	8.66 ± 2.36	8.74 ± 2.54	8.71 ± 1.13
Moisture	71.37 ± 2.85	70.92 ± 3.86	71.67 ± 3.23	71.50 ± 7.33	71.24 ± 4.97	71.63 ± 3.40

All data are expressed as the mean ± SE (*n* = 4).

**Table 5:** Fatty acid composition in the muscle of *C. quadricarinatus* fed diets with different lipid levels (% of total fatty acids).

Parameters	Experimental diets					
	L0	L2	L4	L6	L8	L10
C14:0	1.79 ± 0.24	2.05 ± 0.28	1.61 ± 0.23	1.62 ± 0.02	2.34 ± 0.30	1.99 ± 0.26
C15:0	0.70 ± 0.16	0.70 ± 0.03	0.77 ± 0.20	0.66 ± 0.08	1.30 ± 0.48	0.69 ± 0.02
C16:0	22.94 ± 0.90	23.24 ± 1.20	23.29 ± 0.65	23.82 ± 0.30	24.79 ± 0.87	22.22 ± 0.25
C17:0	2.09 ± 0.24	1.82 ± 0.14	1.93 ± 0.07	1.61 ± 0.15	1.63 ± 0.06	1.68 ± 0.14
C18:0	9.80 ± 0.56	9.70 ± 0.41	10.06 ± 0.35	9.62 ± 0.43	10.36 ± 0.03	9.88 ± 0.53
∑SFA^a^	37.32 ± 0.62	37.51 ± 1.54	37.65 ± 0.79	37.33 ± 0.83	40.41 ± 0.63	36.46 ± 0.45
C16:1	2.34 ± 0.17	2.31 ± 0.28	2.69 ± 0.41	2.35 ± 0.16	2.17 ± 0.18	2.01 ± 0.20
C18:1n-9	24.53 ± 0.25	24.24 ± 0.60	24.30 ± 0.41	24.55 ± 0.31	22.54 ± 1.77	25.14 ± 0.24
∑MUFA^b^	26.87 ± 0.32	26.55 ± 0.51	26.98 ± 0.28	26.90 ± 0.43	24.71 ± 1.60	27.15 ± 0.33
C18:3n-3	1.81 ± 0.09	1.93 ± 0.27	1.63 ± 0.27	1.71 ± 0.13	1.32 ± 0.21	1.65 ± 0.21
C20:5n-3	4.97 ± 0.29	5.89 ± 0.49	5.48 ± 0.49	5.09 ± 0.39	5.74 ± 0.54	6.50 ± 1.15
C22:6n-3	0.80 ± 0.03	1.05 ± 0.22	1.80 ± 0.54	1.28 ± 0.34	0.93 ± 0.07	1.31 ± 0.36
∑n-3PUFA^c^	7.58 ± 0.22	8.87 ± 0.09	8.90 ± 0.52	8.08 ± 0.31	7.99 ± 0.50	9.76 ± 1.07
C18:2n-6	21.73 ± 0.81	20.16 ± 2.76	18.74 ± 2.46	21.08 ± 1.36	16.77 ± 0.78	20.13 ± 1.96
C20:4n-6	5.62 ± 0.13	5.82 ± 0.65	6.45 ± 1.14	5.48 ± 0.25	5.22 ± 0.36	5.46 ± 0.77
∑n-6PUFA^d^	27.35 ± 0.76	25.98 ± 2.11	25.18 ± 1.38	26.57 ± 1.12	21.99 ± 0.73	25.60 ± 1.33
∑PUFA^e^	34.92 ± 0.55	34.85 ± 2.02	34.09 ± 1.07	34.65 ± 1.21	29.98 ± 1.00	35.35 ± 0.84
n-3/n-6PUFA^f^	0.28 ± 0.02	0.35 ± 0.03	0.36 ± 0.04	0.31 ± 0.02	0.36 ± 0.02	0.39 ± 0.06

^a^∑SFA: saturated fatty acids: C14:0, C15:0, C16:0, C17:0, and C18:0. ^b^∑MUFA: monounsaturated fatty acids: C16:1 and C18:1n-9. ^c^∑n-3 PUFA: omega 3 polyunsaturated fatty acids: C18:3n-3, C20:5n-3, and C22:6n-3. ^d^∑n-6 PUFA: omega 6 polyunsaturated fatty acids: C18:2n-6 and C20:4n-6. ^e^∑PUFA: polyunsaturated fatty acids: C18:3n-3, C20:5n-3, C22:6n-3, C18:2n-6, and C20:4n-6. ^f^n-3/n-6 PUFA: omega 3 polyunsaturated fatty acids: omega 6 polyunsaturated fatty acids. All data are expressed as the mean ± SE (*n* = 3). Different letters indicate significant differences between groups (*P* < 0.05).

**Table 6:** Predicted functions of gut microbiota in *C. quadricarinatus* fed diets with different lipid levels. KEGG level 1 and level 3 are listed following the significantly altered pathways.

KEGG level	KEGG pathway	*P* value		
		L0 vs L6	L0 vs L10	L6 vs L10
1	*Metabolism*			
3	Retinol metabolism	0.69	0.04	0.05
3	Secondary bile acid biosynthesis	0.29	0.01	0.02
3	Ubiquinone and other terpenoid-quinone biosynthesis	0.77	0.02	0.01
3	Glycosyltransferases	0.42	0.01	0.01
1	*Cellular processes*			
3	Cytoskeleton proteins	0.26	0.01	0.01
1	*Organismal systems*			
3	Mineral absorption	0.89	0.04	0.04
3	Adipocytokine signaling pathway	0.49	0.03	0.02
3	Antigen processing and presentation	0.68	0.03	0.68
1	*Environmental information processing*			
3	Bacterial toxins	0.68	0.01	0.32
3	Transporters	0.53	0.02	0.24

*P* < 0.05 indicated a significant difference.

## Data Availability

The raw sequencing of gut microbiota was submitted to GenBank with the accession number SRP305670. All other data can be found in the manuscript.
